# Understanding the human gut microbiome: from composition to disease association

**DOI:** 10.3389/frmbi.2026.1717288

**Published:** 2026-04-22

**Authors:** Muneerah Abdullah Alali, Amal Bakr Shori

**Affiliations:** King Abdulaziz University, Faculty of Science, Department of Biological Sciences, Jeddah, Saudi Arabia

**Keywords:** dysbiosis, gut microbiota, host-microbiota interaction, metabolic diseases, microbial metabolites

## Abstract

The human gut microbiota is critical for regulating host metabolism, immune responses, epithelial integrity, and systemic homeostasis, and disturbance has been linked to metabolic, inflammatory, and immune-mediated illnesses. Despite significant advances in microbiome research, the interpretation of gut microbiota-disease relationships is still limited by an overreliance on taxonomic profiling and observational study designs, which frequently overlook functional, strain-level, and mechanistic aspects of host-microbiota interactions. Growing research suggests that microbial functional capacity, metabolic activity, and ecological features such as resilience and functional redundancy are better markers of gut health than compositional measurements alone. Nonetheless, significant inter-individual variability, methodological heterogeneity, and dependence on fecal-based analysis continue to limit reproducibility and causal inference across studies. This review integrates current evidence on gut microbiota composition, functional features, and important influencing variables, while emphasizing mechanistic linkages between microbial dysbiosis and major human illnesses, filling significant conceptual gaps in modern microbiome research.

## 1 Introduction

The human gastrointestinal tract contains a dynamic complex of microorganisms known as the gut microbiota (Thursby and Juge, 2017). This ecosystem is made up of around one thousand microbial species, including bacteria, viruses, archaea, and unicellular eukaryotes, and is believed to include nearly one hundred trillion microbial cells, greatly outnumbering human cells and genes (Lagier et al., 2016; Wang et al., 2017). These microbes inhabit many body sites of the human body, but the gut is the most densely populated and metabolically active microbial location.

The gut microbiota is known as a “vital organ” because of its extensive bidirectional contact with other organs via neurological, endocrine, humoral, immunological, and metabolic pathways (Afzaal et al., 2022). This microbial population is critical to maintaining host homeostasis by controlling important physiological activities including metabolism, immunological responses, epithelial barrier integrity, inflammation, and hematopoiesis (Ahlawat et al., 2021). In physiological terms, the gut microbiota supports human health by regulating nutrient metabolism and energy balance with bioactive metabolites like short-chain fatty acids, as well as shaping immune maturation and tolerance to maintain controlled inflammatory responses (Agus et al., 2021; De Vos et al., 2022; Van Hul et al., 2024a). In parallel, the gut microbiota maintains intestinal epithelial integrity, regulates hematopoietic processes, and enables gut-organ communication, serving as an integrated regulator of host physiological homeostasis (Hou et al., 2022; Van Hul et al., 2024a). Such alterations, commonly described as gut dysbiosis, may include changes in microbial diversity, relative abundance, or metabolic activity, altering host-microbe homeostasis and contributing to disease pathogenesis (Lloyd-Price et al., 2016; Philips et al., 2022).

Host-microbe interactions are critical to both health maintenance and disease development. Diet, age, genetics, lifestyle, and environment all have an impact on the gut microbiota’s diversity and composition. Among these factors, diet plays a significant role in influencing microbial structure and function (Shehbaz et al., 2024). Beyond these extrinsic factors, the gut microbiota helps to maintain microbial homeostasis, which is described as a stable balance of bacteria in the human gastrointestinal tract. Although taxonomic composition varies over time and between individuals, functional redundancy allows the ecosystem to maintain general homeostasis (Sommer et al., 2017; Fassarella et al., 2021). In this context, the gut microbiome’s functional capacity reflects the collective metabolic and signaling potential encoded within the microbial community, which is more preserved across individuals than microbial species composition (Turnbaugh et al., 2008; Visconti et al., 2019a).

A growing body of research has demonstrated that the gut microbiota has a substantial influence on immunological function, energy metabolism, and body weight, as well as being associated with obesity-related illnesses (Piccioni et al., 2022). Beyond metabolic health, disruptions in gut microbial communities and their metabolic products have been linked to a wide range of systemic diseases, including non-alcoholic fatty liver disease (NAFLD), inflammatory bowel disease (IBD), hepatocellular carcinoma, cardiovascular disease (CVD), alcoholic liver disease (ALD), chronic kidney disease (CKD), and cirrhosis (Jansen et al., 2021; Toumi et al., 2021; Wang et al., 2021; Zhou et al., 2021; Philips et al., 2022; Alali and Shori, 2025). For example, recent human cohort studies have identified disease-specific changes in gut microbial composition and functional pathways linked to metabolic diseases (Wu et al., 2020; Toumi et al., 2021). Wu et al (Wu et al., 2020). found distinct gut microbiota signatures associated with impaired glucose metabolism among individuals with prediabetes and type 2 diabetes. In contrast, Aron-Wisnewsky et al (Aron-Wisnewsky et al., 2020). found characteristic microbial and metabolic profiles associated with non-alcoholic fatty liver disease, implying a role for microbiota-derived metabolites in disease-associated metabolic disturbances.

Recent developments in gut microbiome research have been enabled by multi-omics techniques that combine metagenomics, metatranscriptomics, and metabolomics to define microbial activity beyond taxonomic composition. For example, Lloyd-Price et al (Lloyd-Price et al., 2019). used integrated multi-omics data to show disease-associated functional shifts in inflammatory bowel disease, revealing metabolic alterations not captured by taxonomic profiles alone. Similarly, Franzosa et al (Franzosa et al., 2018). discovered that microbial metabolic pathways are more conserved between individuals than species composition, emphasizing the importance of functional ability in establishing gut microbiome homeostasis.

To address the long-standing difficulty of distinguishing between causality and correlation, causal inference and ecological modeling methodologies are increasingly being used in microbiome studies. Sanna et al (Sanna et al., 2019). used Mendelian randomization to establish causal relationships between gut microbial characteristics and metabolic parameters, indicating that microbiota-associated metabolites may directly contribute to disease risk. In parallel, ecological network analysis have indicated that disturbances in microbial interaction networks, rather than changes in individual microbial species, can destabilize gut ecosystems and induce dysbiosis, as reported by Coyte et al (Coyte et al., 2015). Therefore, this review aims to provide an extensive and integrated overview of the human gut microbiota, with an emphasis on its composition, functional aspects in health, and the important intrinsic and extrinsic factors influencing its structure across the lifespan. Furthermore, this review focuses on selected mechanistic pathways linking gut microbiota dysbiosis to major human diseases, highlighting host-microbiota interactions, microbial functional alterations, and emerging evidence that distinguishes correlation from causation. Given the breadth and complexity of microbiome research, particular emphasis is placed on certain mechanistic pathways supported by robust evidence from human studies and multi-omics approaches.

## 2 Research strategy

Relevant literature for this review was identified through searches of major scientific databases, including PubMed, Web of Science, and Google Scholar. Studies were selected based on their relevance to the topic, with particular emphasis on major human diseases and host–microbiota interactions. Priority was given to research providing mechanistic insights into microbiota-associated processes, especially those supported by human data and integrative multi-omics approaches. In addition, recent findings, well-established evidence, and consistency across independent studies were considered to strengthen the interpretation of functional and causal relationships within the gut microbiota. Due to the breadth, complexity, and rapid expansion of the microbiome field, this review adopts a narrative and integrative approach rather than a formal systematic methodology. Accordingly, this approach may not capture all relevant studies or emerging associations, and the selection process may be subject to inherent bias. Furthermore, variations in study design, population heterogeneity, and methodological differences across microbiome research may limit the comprehensiveness and generalizability of the conclusions presented.

## 3 Gut microbiota composition

Beyond classical taxonomic descriptions, the gut microbiota is increasingly considered a dynamic biological ecosystem rather than a static collection of microbial species (Lloyd-Price et al., 2019). Its composition is determined by niche-specific physicochemical circumstances, metabolic interdependence, and functional specialization among microorganisms. Recent multi-omics studies have shown that similar microbial taxonomic structures can be associated with significantly different metabolic activities, demonstrating that microbial functional capacity and metabolic potential are frequently more informative than taxonomic identity alone when defining gut microbiota composition (Franzosa et al., 2019).

Strain-level heterogeneity is increasingly recognized as a significant determinant of gut microbiota composition and function (Zhao et al., 2019). Distinct strains of the same microbial species can have substantial differences in genomic content and metabolic capabilities, resulting in distinct impacts on host-microbe interactions and immune regulation. Advances in shotgun metagenomics and strain-resolved studies have shown that functional features, rather than taxonomic identification, better capture gut microbes’ ecological functions (Franzosa et al., 2018). As a result, gut microbiota composition is increasingly determined by collective functional potential and metabolic outputs rather than the presence or absence of taxa. This strain-level functional diversity contributes to adaptability and resilience of the gut microbial ecosystem.

Furthermore, the composition of the gut microbiota varies significantly between individuals, even among healthy individuals. Large population-based investigations, such as those conducted by Falony et al (Falony et al., 2016), have demonstrated that gut microbial community differs significantly across people due to host-related variables such as genetics, long-term nutrition, age, and environmental exposure. Furthermore, gut microbiota patterns are influenced by methodological determinants. For example, Sinha et al (Sinha et al., 2015), demonstrated that DNA extraction procedures and sequencing methodologies had a considerable impact on identified microbial composition and relative abundance estimations, emphasizing the need of interpreting compositional results within a technical context.

**Figure 1:**
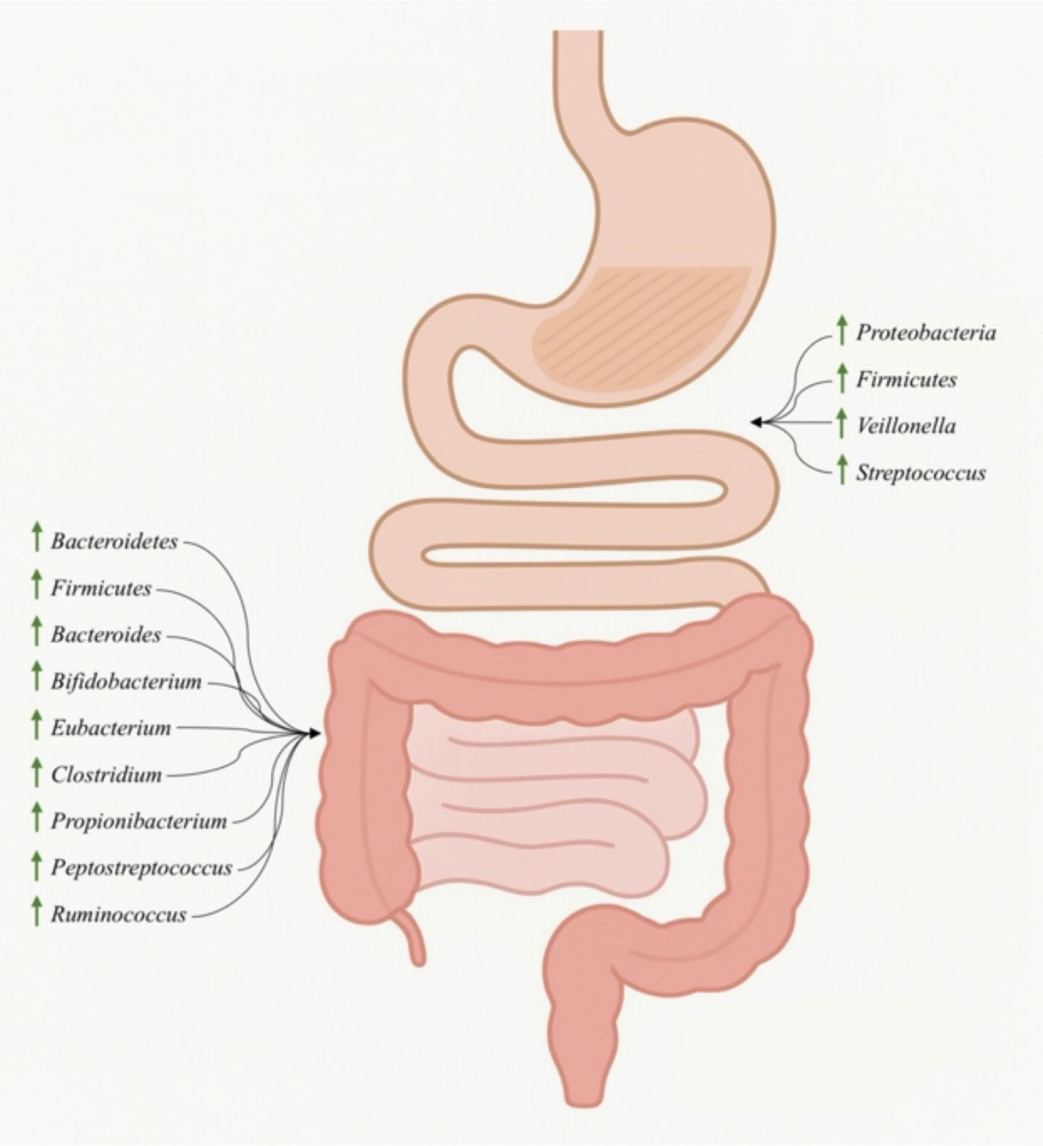
Regional distribution of major gut microbial taxa along the human gastrointestinal tract. ↑ Arrows indicate the relative enrichment or predominance of a specific microbial group in each gastrointestinal region.

The composition of the gut microbiota changes significantly along the gastrointestinal system due to physiological variations such as pH, oxygen availability, substrate accessibility, transit duration, and host secretions (Flint et al., 2012). These variables provide unique microenvironments that structure microbial density, diversity, and dominating taxa across various intestinal segments (Walter and Ley, 2011). **Figure 1** shows that microbial richness and diversity are low in the stomach and small intestine, where acidic conditions, bile exposure, and fast transit promote facultative anaerobes. In contrast, the colon is an anaerobic, nutrient-rich environment that supports dense and varied microbial communities dominated by obligate anaerobes (Walter and Ley, 2011). Spatial and mucosa-related microbiome investigations reveal that gut microbial populations are not evenly dispersed over the gastrointestinal tract, but rather substantially affected by intestinal location, indicating persistent ecological niches linked with each gut region (Zmora et al., 2018).

The stomach and upper small intestine (duodenum, jejunum, and ileum) exhibit low microbial density (10³-10^7^ cells/g tissue) due to extreme conditions such as low pH, bile salts, digestive enzymes, and short transit time. Nonetheless, *Proteobacteria* and *Firmicutes* are predominant in this region, with *Veillonella* and *Streptococcus* being prominent taxa (**Figure 1**) (Aidy et al., 2015; Martinez-Guryn et al., 2018).

In contrast, the large intestine-including the cecum, colon (ascending, transverse, descending, and sigmoid), and rectum-provides an anaerobic environment in which obligate anaerobes can flourish (Eckburg et al., 2005). *Bacteroidetes* and *Firmicutes* dominate the bacterial community, accounting for more than 90% (**Figure 1**) (Arumugam et al., 2011). The number and ratio of these phyla vary by individual and are affected by age, nutrition, and health state. *Bacteroides*, *Bifidobacterium*, *Eubacterium*, *Clostridium*, *Propionibacterium*, *Peptostreptococcus*, and *Ruminococcus* are common genera in the colon, with densities reaching up to 10^9^ cells/g of colonic tissue (**Figure 1** (Mariat et al., 2009).

In addition to bacterial communities, the gut microbiota includes additional biologically active components that help to maintain ecosystem structure and functionality. The gut virome, which is composed of bacteriophages, regulates bacterial population dynamics and shapes microbial community composition through predation and horizontal gene transfer (Shkoporov and Hill, 2019). The gut mycobiome, although comprising a small proportion of total microbial biomass, has been demonstrated to have substantial immunomodulatory effects and impact host inflammatory responses (Iliev and Leonardi, 2017). Furthermore, gut-associated archaea, particularly methanogenic taxa like *Methanobrevibacter* species, play a role in hydrogen metabolism by utilizing bacterial fermentation byproducts, indirectly influencing fermentation efficiency and overall microbial metabolic activity (Gaci et al., 2014).

Although overt gastrointestinal pathogens account for less than 0.1% of the gut microbiota in healthy individuals, they remain clinically relevant. Primary pathogens, including *Salmonella enterica*, *Vibrio cholerae*, and *Campylobacter jejuni* are usually absent or in extremely low prevalence. Opportunistic pathogens, such as *Escherichia coli* and *Bacteroides fragilis*, can be found in healthy persons, accounting for ≥ 0.1% of the microbiota in a subset of individuals, depending on host and environmental conditions. While generally innocuous under normal conditions, these taxa can cause dysbiosis and gastrointestinal illness if their abundance increases or host immunity is impaired (Hollister et al., 2014). Overall, the spatial and compositional diversity of the gut microbiota demonstrates the ecological complexity and functional specialization of microbial niches along the gastrointestinal system.

## 4 Characteristics of a healthy gut microbiota

Defining a “healthy” gut microbiota remains a major challenge in microbiome research, as no single microbial taxonomic composition can be universally associated with health across individuals, largely due to pronounced inter-individual heterogeneity driven by host genetics, age, nutrition, lifestyle, and environmental variables (Lloyd-Price et al., 2016). Identifying the hallmarks of a healthy gut microbiota is crucial to comprehending its function in preserving host health and averting illness. Numerous ecological and biological traits rather than single compositional metrics, have been proposed as indicators of gut health.

In metabolic terms, the capacity of the gut microbiota to convert dietary substrates into bioactive compounds that promote host homeostasis is what defines a healthy gut microbiome. The gut microbiota influences intestinal function, immunological control, energy metabolism, and the biochemical profile of the diet through its metabolic activity (Cardona and Roman, 2022). Short-chain fatty acids, bile acids, branched-chain amino acids, trimethylamine N-oxide, and compounds derived from tryptophan are important metabolites generated by the microbiota that play a key role in mediating host–microbiota communication and regulating physiological processes (Agus et al., 2021). Based on current evidence, **Table 1** summarizes the most often reported features of a healthy gut microbiota, emphasizing biological indicators as well as their potential mechanistic implications.

**Table 1:** Overview of biological markers and mechanistic pathways characterizing a healthy gut microbiota.

Characteristics	Definition/Significance	Mechanisms	References
Diversity	Higher microbial diversity is often associated with improved immunological regulation and resilience to disturbance.	Promotes colonization resistance and resilience through functional redundancy and niche competition.	(Roager et al., 2016; Mohr et al., 2020)
Composition	Focuses on specific taxa and their relative abundance, such as the *Firmicutes/Bacteroidetes* ratio and the presence of beneficial or pathogenic species, with implications for metabolic health, obesity, and inflammation.	Specific taxa influence host metabolism and immunity; an imbalance can result in inflammation or dysbiosis.	(Ley et al., 2006; Duncan et al., 2008; Khachatryan et al., 2008; Donowitz et al., 2019a; Li et al., 2021; Van Hul et al., 2024b)
Functionality	Refers to microorganisms’ functional capabilities, such as metabolite synthesis and enzymatic activity, irrespective of species diversity.	Shared metabolic pathways across species allow for stable gut function despite compositional changes.	(Lozupone et al., 2012; Visconti et al., 2019b; Tian et al., 2020)
Metabolites	Short-chain fatty acids, bile acids, and tryptophan metabolites contribute to immune regulation, energy supply, and mucosal integrity.	SCFAs improve barrier function and immunological modulation, while tryptophan metabolites affect interleukin-22 and antioxidant responses.	(De Vos et al., 2022; Van Hul et al., 2024b)
Strain specificity	Different strains of the same species may have opposite effects—some are probiotic, while others are harmful (e.g. *E. coli*).	Strain-specific gene expression influences inflammation (for example, PSA from Bacteroides fragilis activates Tregs).	(Blandford et al., 2019; Pujo et al., 2021; Enciso-Martínez et al., 2022; Tran et al., 2024; Hu et al., 2025)
Gas production	Microbial byproducts such as methane and hydrogen sulfide production have been associated with intestinal motility and IBS subtypes.	Methane slows transit time (IBS-C), H2S increases permeability (IBS-D); reflects microbial fermentation balance.	(Villanueva-Millan et al., 2022; Van Hul et al., 2024b)
pH level	A colonic pH range of 5.5–7 is ideal for beneficial bacteria and digestive enzyme performance.	SCFA synthesis lowers pH, which inhibits pathogens while promoting acid-tolerant beneficial bacteria.	(Van Hul et al., 2024b)
Inflammation markers	Low levels of fecal calprotectin and lactoferrin indicate low intestinal inflammation and good gut health.	Inflammatory markers indicate mucosal immune activity, which is commonly higher in dysbiosis-related illnesses.	(Pierre et al., 2021)
Resilience	The microbiota’s ability to withstand or recover from perturbations such as antibiotics or diet changes.	Microbial functions are redundant, and adaptive gene expression restores microbial balance following interruption.	(Van Hul et al., 2024b)

*SCFA, Short chain fatty acid, PSA, Polysaccharide A, Tregs, Regulatory T cells, IBS, Irritable bowel syndrome, IBS-C, Irritable bowel syndrome with constipation, IBS-D, Irritable bowel syndrome with diarrhea.

Although notable exceptions exist, higher microbial diversity is frequently associated with favorable health outcomes (Roager et al., 2016; Mohr et al., 2020). Certain compositional metrics, such as the *Firmicutes/Bacteroidetes* ratio, have been thoroughly examined; however, their interpretation as universal health markers remains contested, emphasizing the need for functional and context-dependent frameworks (Ley et al., 2006; Khachatryan et al., 2008). Functional characteristics, such as similar metabolic pathways despite compositional variances, highlight the need to determine functional redundancy (Visconti et al., 2019b; Tian et al., 2020). Functional redundancy refers to the ability of phylogenetically distinct microbial taxa to perform overlapping biological functions, allowing core metabolic activities to be conserved despite significant inter-individual variation in gut microbiota composition (Huttenhower et al., 2012; Moya and Ferrer, 2016; Vieira-Silva et al., 2016).

Beyond functional redundancy, a healthy gut microbiota is increasingly defined by coordinated immunological crosstalk between microbial communities and the host, as well as structured microbial metabolic networks that support cross-feeding and system-level stability (Belkaid and Hand, 2014; Coyte et al., 2015). These higher-order traits cannot be fully captured by taxonomic profiling and are best characterized utilizing integrative multi-omics techniques that relate microbial composition, functional activity, and host responses (Hasin et al., 2017).

Despite its widespread use, the *Firmicutes/Bacteroidetes* (F/B) ratio has limited reliability as a universal biomarker of gut health, owing to significant inter-individual variability and inconsistent associations across studies (Ley et al., 2006; Duncan et al., 2008; Khachatryan et al., 2008). Although variations in this ratio have been seen in metabolic and gastrointestinal illnesses, its interpretation remains largely context dependent. Similarly, taxonomic categories like enterotypes can give helpful population-level insights but may oversimplify the gut microbiome’s dynamic and multifactorial character (Arumugam et al., 2011; Wu et al., 2011; Roager et al., 2014; Vandeputte et al., 2016). At the lower taxonomic levels, some genera, such as *Bifidobacterium* and *Lactobacillus*, are usually associated with beneficial activities; nevertheless, these effects are highly strain-specific and context-dependent and may exhibit neutral or even adverse effects (Donowitz et al., 2019b; Zheng et al., 2020; Li et al., 2021). Collectively, these findings emphasize the limits of using compositional indicators alone to determine gut microbiota health (Van Hul et al., 2024a).

Beyond species-level classification, emerging evidence emphasizes the importance of strain-specific functional traits in defining a healthy gut microbiota. Strain-specific functions refer to functional changes across strains of the same microbial species caused by variations in gene content within the accessory genome, which are influenced by gene gain, loss, and horizontal transfer (Medini et al., 2005). These genomic differences can have distinct metabolic, immunomodulatory, or pathogenic effects, making species identification insufficient for predicting host outcomes (Bisanz et al., 2020). Consistent with this concept, recent clinical evidence highlights the health relevance of strain-level heterogeneity, as different probiotic strains have been demonstrated to significantly affect gut microbiome development and functional interactions with the host, even within the same species (Beck et al., 2022).

Furthermore, metabolite profiling, notably the amounts of short-chain fatty acids and tryptophan-derived metabolites, provides valuable insight into microbial activity and host-microbiota interaction (De Vos et al., 2022). Additional parameters, such as luminal pH and microbial gas production, have attracted increasing attention, particularly in gastrointestinal disorders like irritable bowel syndrome (IBS), where methane and hydrogen sulfide levels have been linked to various clinical subtypes (Villanueva-Millan et al., 2022). Finally, strain-specific effects and inflammatory markers such as fecal calprotectin highlight the importance of precision in microbial characterization and disease interpretation (Pierre et al., 2021; Hu et al., 2025).

When considered collectively, these characteristics provide a conceptual framework for understanding gut microbial health that extends beyond individual compositional metrics. Rather than defining health in terms of taxa or ratios, this integrated view focuses on functional capacity, metabolic outputs, and host-microbiota interactions, all which influence gut ecosystem stability. Such a framework highlights the value of integrative approaches for understanding the complex interplay between microbial composition, function, and host health.

## 5 Factors influencing the gut microbiota

There are numerous environmental, physiological, and social factors that affect the gut microbiota of humans. These factors begin to shape microbial colonization during early life and continue to exert effects throughout the lifespan. Understanding these determinants is essential for explaining inter-individual differences in microbiome composition across age groups. Mode of delivery, infant feeding practices, dietary patterns, age, exposure to antibiotic and other pharmaceutical, and lifestyle-related factors such as physical activity and psychological stress are well-recognized predictors of gut microbiota composition (Kumbhare et al., 2019). The gut microbiota is extremely sensitive to these factors, and its dynamic interactions with the host play crucial roles in immune regulation, metabolic function, and disease susceptibility. From an ecological perspective, the gut microbiota can be considered as a resilient ecosystem capable of maintaining essential functional outputs despite recurring internal and extrinsic disruptions, owing mostly to functional redundancy among microbial communities (Coyte et al., 2015).

These determinants are roughly divided as intrinsic (host genetics, age, and physiological condition) and extrinsic (diet, drugs, environmental exposures, and lifestyle) (**Table 2**). In addition to these factors, host genetic background, exposure to environmental pollutants, socioeconomic status, and circadian rhythm regulation have been identified as important modulators of gut microbiota composition due to effects on immune function, metabolic regulation, and microbial niche availability. **Table 2** gives a complete summary of the primary factors impacting gut microbiota during life, summarizing the accompanying microbial alterations.

**Table 2:** Major factors influencing gut microbiota across the lifespan and their associated microbial changes.

Factor	Factor type	Age/Condition	Major effects on gut microbiota	References
Host genetics	Intrinsic	Adults	Host genetics associated with gut microbiota composition; higher similarity in monozygotic twins than dizygotic twins. Specific taxa (e.g., *Christensenellaceae*) showed significant heritability and were enriched in lean individuals	(Goodrich et al., 2014)
Vaginal delivery	Intrinsic	Newborns	*↑ Lactobacillus, Bacteroides, Prevotella –* early colonization with maternal vaginal microbiota	(Nagpal et al., 2016; Moore and Townsend, 2019)
Cesarean delivery	Intrinsic	Newborns	↑ *Clostridium difficile* and *Staphylococcus;* ↓ *Bacteroides*, *Bifidobacteria* – delayed colonization, exposure to skin/hospital microbiota	(Nagpal et al., 2016)
Aging	Intrinsic	Elderly	↑ *Proteobacteria; enrichment of Akkermansia in centenarians*; ↓ *Bifidobacteria*, *Bacteroides*, SCFA producers; *Firmicutes*/*Bacteroidetes* ratio shifts with age	(Mariat et al., 2009; Biagi et al., 2010; Odamaki et al., 2016; Vaiserman et al., 2020; López-Otín et al., 2023)
Breastfeeding	Extrinsic	First months of life	↑ *Bifidobacterium*, *Lactobacillus*; ↓ *Proteobacteria* – enhanced SCFA production and immune maturation	(Stewart et al., 2018; Ma et al., 2020)
Formula feeding	Extrinsic	First months of life	↑ *Proteobacteria*, *Clostridium* spp. – lower microbial diversity	(Stewart et al., 2018)
Weaning	Extrinsic	9–36 months	↑ *Clostridium leptum*, *Eubacterium hallii*, *Roseburia, Firmicutes*; ↓ *Actinobacteria* – transition to adult-like microbiota	(Bergström et al., 2014; Odamaki et al., 2016)
Diet	Extrinsic	All ages	• Plant-based/Mediterranean: ↑ *Bacteroidetes*, microbial diversity, and SCFA production, ↓ *Proteobacteria* • Western diet: ↑ *Proteobacteria* (inflammation-associated); ↓ diversity, SCFAs • Omnivorous diets: ↑ *Ruminococcus* spp.	(De Filippo et al., 2010; De Filippis et al., 2016; Mitsou et al., 2017; Garcia-Mantrana et al., 2018)
Antibiotics	Extrinsic	All ages	↑ *Enterococcus faecalis; increased risk of Clostridioides difficile infection*; ↓ diversity; shifts in *Firmicutes/Bacteroidetes* ratio	(Coman and Vodnar, 2020; Ramirez et al., 2020)
Other medications (e.g., PPIs, statins)	Extrinsic	Adults/Elderly	↑ Actinomycetaceae and *Staphylococcus*; increased infection susceptibility; ↓ *Bifidobacteriaceae*, *Ruminococcaceae*, and *Lachnospiraceae*	(Falony et al., 2016; Imhann et al., 2017; Hou et al., 2022; Zhang et al., 2023)
Environmental pollutants (air pollution)	Extrinsic	Children	↑ *Rikenellaceae* and *Terrisporobacter;* ↓ *Fusicatenibacter* during smog days in healthy children	(Zheng et al., 2020)
Socioeconomic status (SES)	Extrinsic	Adults	↑ *Prevotella copri*, *Catenibacterium*; ↓ *Dysosmobacter welbionis*	(Kwak et al., 2024)
Physical activity	Extrinsic	Active adults/Elderly	↑ *Akkermansia muciniphila*, *Actinobacteria*, ↓ *Cyanobacteria*, *Proteobacteria* – improved gut barrier and metabolism	(Clarke et al., 2014; Munukka et al., 2018; Zhu et al., 2020)
Stress & Smoking	Extrinsic	All ages	↑ *Escherichia coli*, *Pseudomonas*, *Prevotella* – pro-inflammatory shifts; ↓ *Lactobacilli*	(Lutgendorff et al., 2008; Conlon and Bird, 2014)
Travel & night-shift work	Extrinsic	Adults/Travelers	Circadian rhythm disruption → long-term dysbiosis, ↓ microbial resilience	(Conlon and Bird, 2014)

* ↑ and ↓ indicate the relative increases or decreases in the abundance of the indicated microbial taxa; PPIs, Proton Pump Inhibitors.

It summarizes how the gut microbiota develops in a dynamic and responsive manner, influenced by both internal and external stimuli throughout life. Birth-related events, such as mode of delivery and early feeding practices, have a significant impact on early colonization, determining whether beneficial genera like *Bifidobacterium* and *Lactobacillus* predominate (Nagpal et al., 2016; Stewart et al., 2018), or whether opportunistic microorganisms like *Clostridium difficile* become established (Moore and Townsend, 2019).

Among the intrinsic factors of gut microbiota composition, host genetic background has emerged as a significant contributor to interindividual heterogeneity in microbial taxa. Large-scale twin studies provide significant evidence that genetic factors influence the gut microbiota, since monozygotic twins have more compositional similarity than dizygotic twins. Goodrich et al (Goodrich et al., 2014). conducted a landmark study on fecal samples from a large twin cohort, demonstrating that microbial species exhibit heritable patterns regardless of common environmental exposure. These findings lend credence to the idea that host genetics selectively influences gut microbiota structure by varying the presence and relative abundance of specific microbial lineages, thereby contributing to the individuality and stability of the human gut ecosystem. In later stages of life, aging is associated with different changes in gut microbiota composition, such as decreasing microbial diversity and modifications in functional microbial profiles. In a large human cohort study, Wilmanski et al (Wilmanski et al., 2021). found that age-related alterations in the gut microbiome result in lower microbial resilience and altered metabolic capacity, even in otherwise healthy elderly people.

Diet remains one of the most important modulators of gut microbiota composition. Plant-based and Mediterranean diets (e.g., high intake of fruits, vegetables, wholes grains, legumes, olive oil, and nuts) have been linked to enhanced microbial diversity and short-chain fatty acid (SCFA) synthesis, which promote metabolic and immunological health (De Filippis et al., 2016; Mitsou et al., 2017). By contrast, Western-style diets (e.g., high consumption of processed foods, refined sugars, saturated fats, and red/processed meats) have been related to reduced microbial diversity and enrichment of pro-inflammatory taxa (De Filippo et al., 2010).

Furthermore, antibiotic use can drastically diminish microbial diversity while promoting the growth of pathogenic species like *Enterococcus faecalis* and *Clostridium difficile*, with long-term consequences (Panda et al., 2014; Coman and Vodnar, 2020). For example, a population-level human metagenomic study demonstrated that antibiotic exposure was associated with a significant decrease in gut microbial diversity and persistent alterations in community composition across cohorts, supporting the long-term impact of antibiotic-associated dysbiosis (Lee et al., 2023). Recovery may necessitate probiotic or nutritional interventions. Other drugs, such as proton pump inhibitors (PPIs), have also been linked to a deleterious influence on gut microbiota (Imhann et al., 2017).

In addition to antibiotics, other commonly administered medications have been proven to influence gut microbial composition. Metformin therapy causes considerable changes in gut microbial community structure and enriches beneficial taxa, implying a direct drug-microbiome interaction (Niu et al., 2024). Likewise, antipsychotic drugs have been linked to significant changes in gut microbiota composition, including shifts in the relative abundance of major bacterial phyla (Bahr et al., 2015). In cancer therapy, immune checkpoint inhibitors interact with baseline gut microbiota composition, where distinct microbial signatures, such as enrichment of *Alistipes* and depletion of *Bacilli* and *Lactobacillales*, have been linked to improved responses to anti-PD-1/PD-L1 (Zhu et al., 2024). Collectively, these drug-induced changes represent ecological perturbations to the gut microbiota. Disruptions in microbial community structure can destabilize ecosystem interactions and influence recovery dynamics, which is consistent with ecological principles of microbiome stability and resilience (Coyte et al., 2015).

Environmental pollution has emerged as a significant extrinsic factor influencing gut microbiota composition in humans. Human observational studies show that exposure to air pollution can cause detectable changes in gut microbial populations, even in otherwise healthy individuals. In a panel investigation evaluating the impacts of smog exposure, Zheng et al (Zheng et al., 2020). found different microbiota alterations in healthy children during smog days compared to clean days, with a reduction in *Fusicatenibacter* and an increase in *Rikenellaceae* and *Terrisporobacter*. These data indicate that ambient air pollution is sufficient to disrupt gut microbial equilibrium, emphasizing environmental contaminants as independent modulators of gut microbiota composition.

In addition, socioeconomic status (SES) has emerged as a major extrinsic factor influencing gut microbiota composition. Lower socioeconomic status (SES) has been linked to altered microbial community structure, including altered β-diversity and differential abundance of specific taxa, such as increased *Prevotella copri* and *Catenibacterium* spp., alongside reduced *Dysosmobacter welbionis* (Kwak et al., 2024). This suggests that socioeconomic conditions may shape gut microbial ecology through diet quality, environmental exposures, and psychosocial stress.

Lifestyle factors, such as circadian rhythm modulation and physical exercise, have a significant impact on gut microbiota composition and function. Thaiss et al (Thaiss et al., 2014). found that gut microbiota composition and function are impacted by host circadian biology, particularly feeding-fasting rhythms. Disruption of circadian alignment, such as genetic clock impairment or ambient jet lag, led in microbial rhythmicity loss and dysbiosis, which was connected to host metabolic dysfunction.

In a complementary manner, physical activity has also been shown to modulate gut microbiota composition. A human investigation on elite athletes found that exercise is associated with distinct gut microbiota compositions. In this study, O’Donovan et al (O’Donovan et al., 2020). classified athletes based on the dynamic and static components of their sports and found that athletes who participated in sports with a moderate dynamic component had higher relative abundances of *Anaerostipes hadrus*, *Streptococcus suis*, and *Clostridium bolteae*. Notably, the enrichment of the butyrate-producing bacterium *A. hadrus* indicates a possible link between moderate-intensity physical activity and favorable functional alterations in the gut microbiome, independent of dietary intake. These findings suggest that repeated lifestyle-related stimuli, such as circadian rhythm modulation and habitual physical activity, may influence not only gut microbiota composition but also the stability of functional interactions over time, which is consistent with ecological principles of microbiome stability and resilience (Coyte et al., 2015).

## 6 Gut microbiota in major human diseases

**Figure 2:**
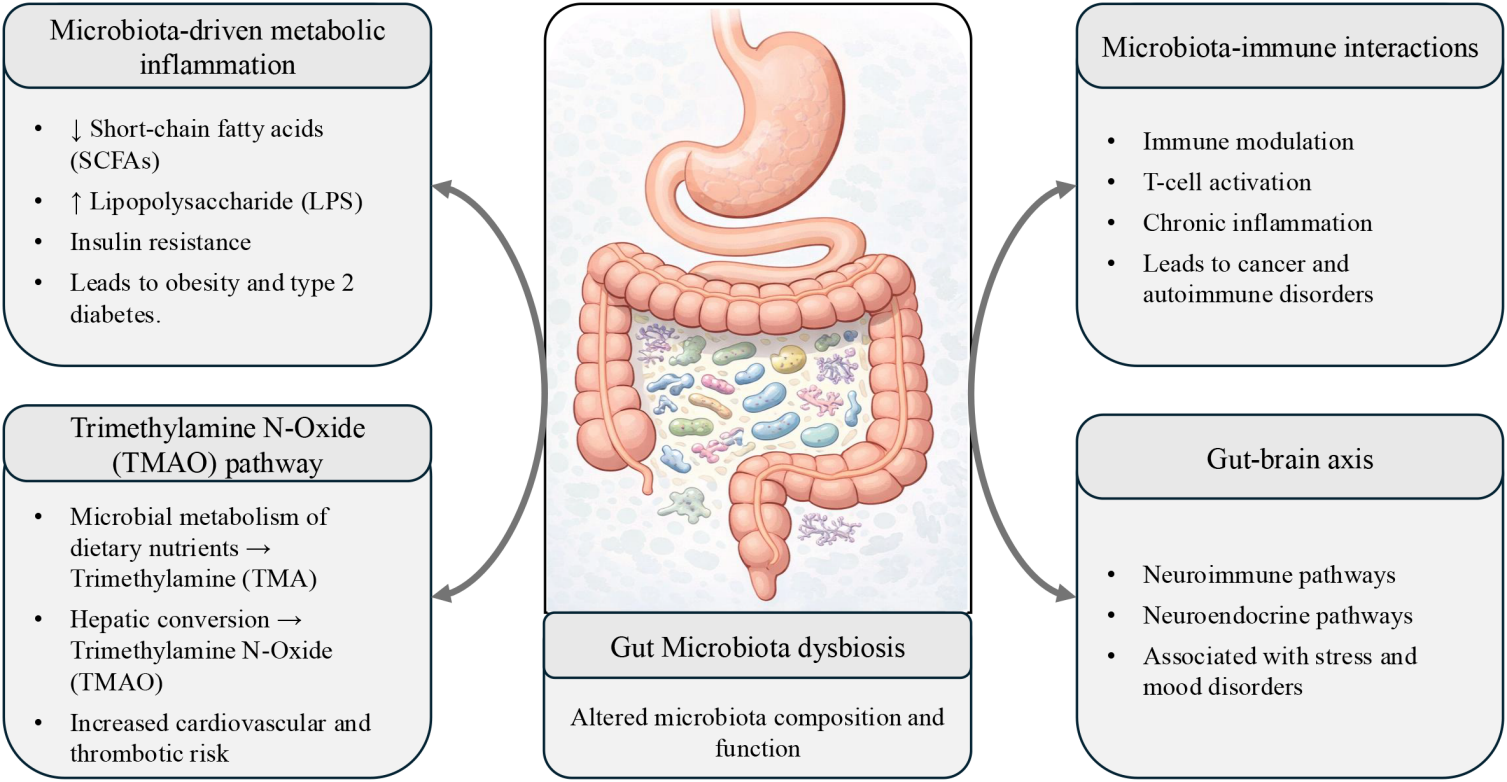
Major mechanistic pathways linking gut microbiota dysbiosis to metabolic, immune, cardiovascular, and neuroendocrine disorders.

The human gut microbiota has emerged as a key regulator of host physiology, with effects reaching far beyond gastrointestinal homeostasis to metabolic, immunological, and neuroendocrine systems. Growing evidence from epidemiological research, experimental animal models, and multi-omics studies shows that disruptions in gut microbial composition and function, commonly referred to as dysbiosis, are intimately associated with the beginning and progression of a variety of chronic diseases (**Figure 2**). While early research focused primarily on disease-associated microbial signatures, more recent studies have begun to unravel causative processes by which the gut microbiota actively contributes to disease pathogenesis rather than just reflecting disease states. These pathways include microbiota-induced metabolic inflammation, host immune response modification, epithelial barrier disruption, and altered microbe-derived metabolite signaling along the gut-organ axis. Consequently, the gut microbiota is now recognized as both an indicator of disease risk and a modifiable therapeutic target, with growing implications for precision medicine and microbiome-based therapies.

### 6.1 Gut microbiome dysbiosis as a causal factor in human disease

Although several studies have found links between altered gut microbial patterns and a variety of human diseases, determining causality remains a major difficulty in microbiome research (Lloyd-Price et al., 2016). Most human studies are observational and cross-sectional, making it difficult to tell whether microbial dysbiosis is a cause or a result of illness progression. Inter-individual variability, as well as confounding factors including diet, medication usage, and environmental exposures, make it difficult to identify a common “healthy” microbiome makeup (Falony et al., 2016). consequently, disease-associated microbial profiles should be evaluated with caution when determining mechanistic roles in pathogenesis. 

To address these constraints, experimental models and longitudinal techniques have proved useful in determining causal links between the gut microbiota alterations and human disease (Fassarella et al., 2021). Evidence from antibiotic-induced microbiota perturbation and fecal microbiota transplantation all show that disease-associated microbial communities can transmit pathological traits to healthy hosts (Marchesi et al., 2016). In humans, controlled fecal microbiota transplantation trials have shown that changing gut microbial composition can influence host metabolic phenotypes, supporting the microbiota’s causal role in metabolic dysregulation (Vrieze et al., 2012). Importantly, contemporary models emphasize that dysbiosis extends beyond taxonomic compositional shifts and instead reflects functional disruption, characterized by altered metabolic capacity, reduced ecological resilience, and impaired host–microbiota homeostasis (Fassarella et al., 2021). Collectively, these findings support the concept of gut dysbiosis as an active driver of disease processes rather than merely a passive biomarker, providing a mechanistic rationale for microbiome-targeted therapeutic strategies (Marchesi et al., 2016).

### 6.2 Microbiota-driven metabolic inflammation and cardiometabolic diseases

Cardiometabolic illnesses, such as obesity, type 2 diabetes, and cardiovascular disease, are increasingly recognized as chronic inflammatory disorders caused by intricate interactions between host metabolism, immune responses, and environmental variables. Human observational and clinical studies show that the gut microbiota influences disease development and progression by modulating energy harvest, lipid metabolism, inflammatory signaling, and intestinal barrier integrity (Tang et al., 2014; Tilg et al., 2020).

The trimethylamine N-oxide (TMAO) pathway is a well-established link between gut microbiota and cardiovascular disease in humans. Dietary nutrients containing choline, phosphatidylcholine, and L-carnitine are degraded by gut microorganisms into trimethylamine (TMA), which is then converted to TMAO in the liver, a process that has been consistently reported in human studies. In large prospective cohorts, elevated circulating TMAO levels have been independently associated with an increased risk of cardiovascular and thrombotic risks, including myocardial infarction and stroke, even after adjustment for established risk factors (Tang et al., 2014). Physiologically relevant concentrations of TMAO directly increase human platelet responsiveness through increased intracellular calcium signaling, providing functional human support for a microbiota–thrombosis axis, according to mechanistic evidence from ex vivo functional studies (Zhu et al., 2016).

Beyond trimethylamine N-oxide (TMAO), a growing body of evidence highlights the critical role of diverse microbiota-derived metabolites in the development of metabolic dysfunction-associated steatotic liver disease (MASLD). Gut dysbiosis contributes to disease progression by disrupting intestinal barrier integrity, facilitating the translocation of microbial products such as lipopolysaccharide (LPS) to enter the portal circulation and activate TLR4-mediated inflammatory signaling pathways (Schnabl et al., 2025). This mechanism increases hepatic inflammation, insulin resistance, and lipid accumulation, accelerating the progression from steatosis to steatohepatitis and fibrosis. In parallel, microbial metabolites such as short-chain fatty acids, bile acid derivatives, ethanol, and phenylacetate modulate host metabolic and immunological pathways, affecting hepatic lipid metabolism and inflammatory responses (Schnabl et al., 2025). Collectively, these methods highlight the critical role of the gut-liver axis and microbiota-derived metabolites in MASLD development.

Microbiota-host interactions also involve enteroendocrine regulation and innate immune activation, in addition to metabolite-driven and endotoxin-mediated pathways. The gut microbiota modulates L-cell activity and glucagon-like peptide-1 (GLP-1) secretion, thereby influencing insulin secretion and metabolic homeostasis (Christ et al., 2019). In parallel, microbiota-derived signals lead to epigenetic remodeling in immune and metabolic cells, perpetuating inflammatory dysregulation (Christ et al., 2019). Activation of the NLRP3 inflammasome in response to metabolic stress, leading to IL-1β-mediated inflammation and insulin resistance (Christ et al., 2019; Donath and Drucker, 2025). These interrelated processes underscore the importance of microbiota-driven endocrine-immune crosstalk in the development and progression of cardiometabolic disorders.

In addition to metabolite-driven pathways, persistent low-grade systemic inflammation in humans and compromised intestinal barrier function have been strongly linked to gut microbiota-associated metabolic inflammation. Increased translocation of microbial components, especially lipopolysaccharide (LPS), into the systemic circulation is linked to dysbiosis-related changes in gut permeability. This phenomenon is correlated with insulin resistance, adipose tissue inflammation, and disturbed glucose homeostasis in people with type 2 diabetes and obesity (Tilg et al., 2020). Recent human studies indicate that sustained exposure to LPS increases metabolic inflammation by activating innate immune signaling pathways, which accelerates the progression of cardiometabolic illness (Fan and Pedersen, 2021). Furthermore, decreased levels of bacteria that produce short-chain fatty acids have been linked to altered metabolic signaling and compromised immune regulation, which increases vulnerability to cardiometabolic illnesses (Fassarella et al., 2021).

### 6.3 Microbiota–immune interactions in immune-mediated diseases

The gut microbiota has a substantial impact on systemic immune responses, influencing a wide range of human diseases, including allergic, inflammation and autoimmune disorders, as well as cancer. Commensal microorganisms interact with the host immune system to control antigen presentation, T-cell activation, and inflammatory signaling pathways, all of which influence immunological surveillance and tolerance (Belkaid and Hand, 2014; Honda and Littman, 2016). Through these processes, the gut microbiota helps to establish immunological tone, thereby modulating susceptibility to disease progression and chronic immune activation (Zitvogel et al., 2018).

At the molecular level, gut microbiota interacts with the host immune system through pattern recognition receptors (PRRs), which recognize microbe-associated molecular patterns (MAMPs). This recognition initiates downstream signaling cascades that regulate innate and adaptive immune responses (Cortés et al., 2025). These interactions are essential for maintaining immunological homeostasis by promoting regulatory pathways and avoid excessive inflammation. Microbiota-derived signals contribute to the differentiation of regulatory T cells (Tregs), which are essential for immune tolerance. Furthermore, gut microbiota supports the intestinal epithelial barrier by modulating tight junction proteins and limiting microbial translocation, which reduces systemic inflammatory responses (Cortés et al., 2025).

Emerging evidence indicate that microbiota-immune interactions go beyond classical inflammatory and autoimmune disorders to other systemic axes. Gut dysbiosis has been associated with dermatological disorders including psoriasis and atopic dermatitis through immune crosstalk, altered short-chain fatty acid production, and increased pro-inflammatory responses (Colica et al., 2026). Similarly, rheumatological diseases have been associated with gut microbiota alterations through mechanisms involving chronic immune activation, increased intestinal permeability, and disruption of Th17/Treg balance, all of which may amplify systemic inflammation (Shen et al., 2025; Colica et al., 2026). Furthermore, the gut-placenta axis has emerged as a relevant pathway, since maternal gut microbiota and its metabolites appear to influence placental homeostasis, morphogenesis, and fetal development (Shen et al., 2025). Together, these findings highlight the broader systemic relevance of microbiota-driven immune modulation across multiple physiological interfaces.

Asthma and other respiratory illnesses have been linked to microbiome disruptions, particularly during infections like bronchitis and pneumonia caused by *Chlamydophila pneumoniae* (Huang and Boushey, 2015). These interactions are assumed to be mediated by immunologically related pathways along the gut-lung axis, with microbiota-derived signals influencing systemic immune modulation and pulmonary inflammatory responses (Budden et al., 2017). Early exposure to beneficial microbial communities has been shown to enhance immune maturation and protect against allergic reactions by reducing excessive allergy-prone inflammatory responses and promoting immune tolerance during early life (Ege et al., 2011). Similarly, gastrointestinal illnesses caused by bacteria like *Campylobacter*, *Salmonella*, *Escherichia coli*, and *Shigella* can cause dysbiosis by inducing intestinal inflammation, disrupting epithelial barrier integrity, and displacing resident commensal microbial communities, collectively altering microbial balance (Josephs-Spaulding et al., 2016).

Studies on immune checkpoint inhibitor (ICI) treatment in cancer patients have shown strong human evidence for microbiota-immune interactions. Gopalakrishnan et al (Gopalakrishnan et al., 2018). found a substantial difference in baseline gut microbiota composition between responders and non-responders in melanoma patients treated with anti-PD-1 drugs. Responders exhibited improved antigen presentation and intratumoral CD8^-^ T-cell infiltration. Similar relationships have been seen across different cancer types, with gut microbiome patterns correlating with treatment success and systemic immune activation (Routy et al., 2018). Importantly, interventional clinical studies have shown that fecal microbiota transplantation (FMT) from immunotherapy responders into patients with refractory melanoma restored responsiveness to anti-PD-1 therapy, which was accompanied by immune reprogramming within the tumor microenvironment (Baruch et al., 2021; Davar et al., 2021).

Beyond cancer, microbiota-immune interactions have been linked to the development of autoimmune and inflammatory illnesses in humans. Dysbiosis has been associated with reduced immunological tolerance and higher inflammatory responses, potentially driven by altered microbial-immune communication and greater exposure to microbial antigens (Belkaid and Hand, 2014; Honda and Littman, 2016). Furthermore, clinical and immunological data in human populations have led to the hypothesis that molecular mimicry between microbial components and host antigens contributes to autoreactive immune responses in autoimmune disorders (Rojas et al., 2018). Collectively, these findings highlight the gut microbiota as a critical regulator of immunological homeostasis, with microbial dysregulation linked to both cancer immunity and autoimmune disease development.

### 6.4 Neuroimmune and neuroendocrine pathways of the gut–brain axis

The gut-brain axis is a bidirectional communication network that connects the gastrointestinal tract to the central nervous system via integrated neuronal, immunological, and endocrine pathways. In humans, emerging data suggests that gut microbiota composition and function impact brain physiology and behavior via influencing immunological signaling, neuroendocrine responses, and microbial metabolite synthesis. Dysbiosis has been linked to altered gut-brain communication, which contributes to neuroinflammation, stress dysregulation, and mood disorders, emphasizing the gut microbiota as a critical regulator of brain-body balance (Cryan et al., 2019; Akram et al., 2024).

Neuroimmune pathways are a significant mechanism underlying microbiota-brain interactions in humans. Human observational and clinical investigations have shown that variations in gut microbiota composition are linked to changes in immunological and metabolic signals that influence brain function and behavior. Large human cohort studies have found links between specific gut microbial profiles, circulating inflammatory and neuroactive metabolites, mood and cognition outcomes, indicating that neuroimmune pathways play a role in gut-brain communication (Valles-Colomer et al., 2019). These findings suggest that immune-mediated signaling, rather than direct microbial translocation, is critical for delivering gut-derived signals to the brain.

In parallel, neuroendocrine pathways play an important role in gut-brain transmission, notably via modulating stress-related hormonal responses. Human clinical research has revealed that changes in gut microbiota composition are related with variations in brain activity and stress processing, emphasizing the link between gut microbiota and neuroendocrine control. Modifying gut microbial communities through dietary or probiotic interventions, for example, has been shown to alter neural activity in brain regions involved in emotion and sensory processing in healthy individuals, indicating microbiota-dependent regulation of neuroendocrine and neural pathways (Tillisch et al., 2013). Neuroimmune and neuroendocrine systems work together to offer complementary pathways for gut-brain communication, strengthening the gut microbiota’s function in regulating neurophysiological and psychological balance.

### 6.5 Therapeutic implications of gut microbiota modulation

Understanding host–microbiota interactions provide a mechanistic foundation for the development of specific microbiota-based therapies. Understanding how specific microbial taxa and microbiota-derived metabolites interact with host metabolic, immune, and epithelial pathways enables a shift from empirical interventions and toward mechanism-driven therapeutic strategies aimed at restoring functional host-microbiota homeostasis rather than broadly altering microbial composition (Agus et al., 2021; Van Hul et al., 2024a).

Dietary interventions are the most well-established and evidence-based technique for modifying the gut microbiota in humans. Controlled human studies show that consuming a variety of plant-based fibers on a regular basis enhances microbial diversity, promotes short-chain fatty acid production, and improves metabolic and inflammatory biomarker levels. Wastyk et al (Wastyk et al., 2021). demonstrated in a randomized dietary intervention trial that a high-fiber diet in healthy adults resulted in measurable changes in gut microbial composition as well as reduced inflammatory markers, indicating a causal link between diet-induced microbial changes and host immune regulation. These findings indicate that nutritional modification of the gut microbiota can have clinically significant impacts on host physiology in humans.

However, emerging evidence suggests that responses to microbiota-targeted dietary treatments are highly individualized. Large-scale human cohort studies have shown that inter-individual diversity in baseline gut microbiota composition has a significant influence on metabolic and glycemic responses to equal food exposures. In a landmark prospective investigation, Zeevi et al (Zeevi et al., 2015). found that tailored dietary predictions including individual microbiome traits predicted postprandial glycemic responses more accurately than generalized dietary recommendations. More recently, population-level investigations have demonstrated that cardiometabolic responses to nutritional interventions vary significantly between individuals due to microbiota-dependent functional variations (Asnicar et al., 2025). Collectively, these findings underscore the limitations of universal dietary guidelines and the importance of microbiota-informed customized nutrition approaches.

Aside from dietary methods, direct microbiota-based therapies offer the most compelling clinical evidence for therapeutic regulation of the gut microbiota in humans. Fecal microbiota transplantation (FMT) has repeatedly shown great clinical efficacy in people with recurrent *Clostridium difficile* infection, which is predominantly caused by antibiotic-induced dysbiosis. A systematic review and meta-analysis of human clinical studies found that clinical cure rates ranged from roughly 84% after single FMT to 91% after repeat FMT at week 8, supporting microbial restoration as a successful therapeutic method in this setting (Baunwall et al., 2020). However, outside this specific indication, human studies exploring microbiota-based interventions have shown more heterogeneous outcomes, underscoring the need for careful patient selection and individualized therapeutic design.

## 7 Future perspectives

Future advances in gut microbiota–targeted therapeutics are expected to prioritize precision and function-based interventions rather than broad microbial manipulation. Integrating multi-omics techniques with host-specific parameters such as genetics, immunological condition, and baseline microbiota composition may allow for more personalized strategies to restore functional host-microbiota homeostasis while conserving microbial resilience. Such an approach is essential to minimize unintended ecological perturbations within the gut ecosystem.

Future research should focus on addressing key unresolved questions, such as determining causality through well-designed longitudinal and interventional human studies, identifying functionally relevant microbial metabolites, and developing robust host-microbiota biomarkers with clinical predictive value. Furthermore, integrating strain-level resolution, spatial microbiome profiling, and standardized multi-omics pipelines will be critical for improving reproducibility and transforming microbiome research into reliable clinical tools for disease risk stratification and therapeutic monitoring.

As microbiota-based therapeutics advance, ethical and safety considerations become increasingly important. Therapeutic alteration of the gut microbiota may pose risks such as long-term ecosystem instability, unintentional dysbiosis, pathogen overgrowth, horizontal gene transfer of antimicrobial resistance genes, and unanticipated immunological or metabolic responses. These concerns highlight the importance of comprehensive safety evaluation, standardized intervention methods, long-term follow-up, and clear informed consent, especially when dealing with vulnerable groups.

## 8 Conclusion

The gut microbiota is increasingly recognized as a dynamic and functionally integrated ecosystem that contributes substantially to host metabolism, immune regulation, epithelial integrity, and overall physiological homeostasis. Growing evidence indicates that gut microbiota-host interactions are influenced not only by microbial composition, but also by strain-specific diversity, functional capacity, metabolic activity, and ecological stability. This shift from simply taxonomic descriptions to functional and mechanistic frameworks has significantly improved our understanding of how microbial dysbiosis contributes to disease susceptibility and development across a wide variety of diseases.

Recent findings show that functional traits and metabolic pathways are frequently more preserved across people than microbial taxa, emphasizing the relevance of ecological concepts like functional redundancy and resilience in maintaining gut microbiota stability. As a result, interpretations based purely on compositional indicators or specific microbial markers are widely seen as insufficient to explain complex host-microbiota interactions or characterize gut health in a physiologically relevant way. Despite these conceptual and methodological advances, current gut microbiota research is limited by the prevalence of observational study designs, significant inter-individual variability influenced by host and environmental factors, methodological inconsistencies across analytical pipelines, and an overreliance on fecal taxonomic profiling, which does not fully reflect strain-level, functional, and spatial microbial dynamics within the gastrointestinal tract. Bridging these mechanistic insights with clinical application will be essential for advancing microbiome-informed diagnostics, improving disease risk stratification, and enabling personalized microbiota-based therapeutic strategies in future clinical practice.
